# Association Between Maternal Vitamin K2 Levels in Late Pregnancy and Newborn Bone Metabolism

**DOI:** 10.1002/fsn3.70363

**Published:** 2025-05-30

**Authors:** Xuejing Liu, Shuo Wang, Han Chen, Nianfeng Qian, Lina Wu, Yingnuo Liu, Zhaoxi Hou, Yueting Bai, Hongqing Jiang

**Affiliations:** ^1^ Department of Gynecology and Obstetrics Haidian Maternal and Child Health Hospital Beijing China

**Keywords:** 25‐hydroxyvitamin D, bone metabolic markers, newborn, pregnancy, vitamin K2

## Abstract

We aimed to explore maternal vitamin K2 levels in late pregnancy and their association with neonatal bone metabolism markers. This study included 197 pregnant women and their matched newborns. Blood samples were collected from the mothers 1–2 days before delivery and from the umbilical cord immediately after birth. Serum vitamin K2 and 25‐hydroxyvitamin D (25‐OHD) levels were measured using liquid chromatography–tandem mass spectrometry. Maternal vitamin K2 levels were categorized into vitamin K2 deficiency (< 0.1 ng/mL) and normal (0.1–0.86 ng/mL) groups. Maternal vitamin K2 deficiency prevalence was 38.6%. Spearman's correlation analysis demonstrated a significant association between maternal vitamin K2 and newborn 25‐OHD levels (*r* = 0.368, *p* < 0.001). Maternal osteocalcin (OC), newborn OC, and newborn parathyroid hormone levels in the vitamin K2 deficiency group were significantly higher than those in the normal group. Univariate and multivariate logistic regression analysis indicated that maternal vitamin K2 and newborn calcium levels were independent risk factors for neonatal 25‐OHD insufficiency. In conclusion, maternal vitamin K2 deficiency is prevalent during late pregnancy and may adversely affect both maternal and neonatal bone metabolism, highlighting the importance of vitamin K2 supplementation during pregnancy.

## Graphical abstract

Vitamin K2 regulates bone metabolism and contributes positively to bone health and mineralization. Vitamin K2 deficiency is prevalent among pregnant women during the late stages of pregnancy and is an independent risk factor for 25‐OHD insufficiency in newborns. It is important to monitor and supplement vitamin K2 levels during pregnancy to promote optimal skeletal development in neonates.

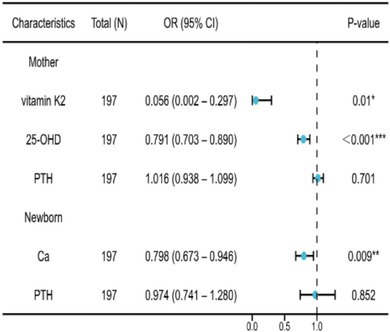

## 1 Introduction

Bone growth, development, and metabolism play crucial roles in the overall well‐being and maturation of newborns (Gómez‐Alonso 2020). During fetal development, the skeletal system relies primarily on the placenta to supply essential minerals, such as calcium (Ca), parathyroid hormone (PTH), osteocalcin (OC), and other mineral components (Sethi et al. 2020). Reportedly, the prevalence of impaired bone development in infants under 1 year of age is as high as 30% and is even higher in early childhood (Tan et al. 2020). Anomalous bone metabolism can lead to skeletal disorders, which, in turn, affect the growth, development, and overall health of newborns (Arnold et al. 2021). Biochemical indicators of bone metabolism include Ca and 25‐hydroxyvitamin D (25‐OHD) (Group, B. M. E 2023). Additionally, markers of bone formation, such as total alkaline phosphatase (ALP) and OC, and markers of regulatory hormones, such as PTH, serve as significant indicators of bone health (Kanis et al. 2021). Consequently, thorough research on bone metabolism in newborns is imperative to comprehend and prevent metabolic bone diseases, such as osteoporosis and rickets, in children.

Vitamin K2 is a lipid‐soluble vitamin distinguished by its menaquinone structure with a polyisoprenoid side chain (Mladěnka et al. 2022). It is naturally present in animal fats, natto, cheese, and fermented foods (Ren et al. 2020). Vitamin K2 regulates bone metabolism and contributes positively to bone health and mineralization by activating OC, a protein that binds Ca to the bone matrix (Li et al. 2019). Li et al. (2019) study demonstrated that vitamin K2 can be used to treat postmenopausal osteoporosis, fractures, and other bone‐related diseases. Hungarian researchers have demonstrated that variations in bone mineral status among 10–12‐year‐old children, evaluated using ultrasound, are associated with optimal vitamin K2 intake (Szmodis et al. 2019). Additionally, vitamin K2 supplementation can enhance bone mineral density and reduce fracture risk in postmenopausal women (Sato et al. 2020). During pregnancy, women experience an increased demand for vitamin K2 due to hormonal changes, fetal growth and development, augmented blood volume, dietary and nutritional factors, and modifications in the gut microbiota (Chatterjee et al. 2023). To date, maternal vitamin K2 levels in late pregnancy and their correlation with neonatal bone metabolism markers remain largely unexplored. Therefore, this study aimed to investigate the association between maternal vitamin K2 levels and newborn bone metabolism markers.

## 2 Materials and Methods

### 2.1 Study Population

In total, 197 pairs of pregnant women and their newborns were recruited for this study between January 2024 and August 2024 at the Beijing Haidian Maternal and Child Health Hospital. Based on the vitamin K2 levels of pregnant women, the study participants were divided into two groups: a vitamin K2 deficiency group (vitamin K2 level < 0.1 ng/mL) and a normal group (vitamin K2 level of 0.1–0.86 ng/mL), following the standards set by MDI laboratory in Germany. The inclusion criteria were singleton pregnancy, full‐term delivery (37–42 weeks), non‐smoking, non‐drinking, complete clinical case notes, and signed informed consent. Participants were excluded if they had conditions that could affect bone or Ca metabolism, such as hyperparathyroidism, renal failure, chronic diseases treated with corticosteroids, preeclampsia, or thyroid dysfunction. Newborns with complex perinatal conditions, including muscular, neurological, and skeletal disorders, were excluded.

### 2.2 Specimen Collection and Liquid Chromatography‐Tandem Mass Spectrometer Method

Venous blood samples (3 mL) were obtained from pregnant women 1–2 days before delivery. The samples were used to examine vitamin K2, 25‐OHD, Ca, ALP, PTH, and OC levels. Umbilical cord blood samples (3 mL) were collected immediately after delivery. Following collection, the samples were centrifuged at 3500 rpm at 4°C for 10 min. The supernatants were transferred to new tubes and stored at 4°C. Subsequently, the blood samples were sent to the Hehe Medical Laboratory for analysis. Serum vitamin K2 and 25‐OHD levels were measured using a liquid chromatography‐tandem mass spectrometer (LCMS‐8050CL, Shimadzu Corporation). Before analysis, blood samples were purified using organic solvents, such as methanol or acetonitrile. An internal standard with chemical properties similar to those of the target compounds was used. The blood samples were analyzed using liquid chromatography–tandem mass spectrometry to measure the peak areas of the target and internal standard substances, including vitamins K2 and 25‐OHD, after adjusting the parameters.

### 2.3 Measurement of Bone Metabolic Markers

Ca concentrations were quantified using inductively coupled plasma mass spectrometry (Thermo Fisher Scientific, Waltham, MA, USA). OC levels were assessed using a Roche Cobase601 electrochemical luminescence instrument (Roche Diagnostics, Mannheim, Germany), whereas PTH levels were measured using a CL2000i chemiluminescent microparticle immunoassay (Mindray, Shenzhen, China). ALP activity was determined using BS800 and its corresponding reagents (Mindray, Shenzhen, China). The above indicators were detected strictly according to the manufacturer's instructions.

### 2.4 Statistical Analysis

All statistical analysis were performed using SPSS version 24.0 (SPSS, Chicago, IL, USA). Data are presented as the mean ± standard deviation ($\bar{X}$ ± SD). Differences between vitamin K2 normal and deficient groups and various clinical parameters were assessed using *t*‐tests or chi‐square tests (*χ* ^2^). Spearman's correlation analysis was conducted to explore the relationship between maternal vitamin K2 levels and neonatal 25‐OHD levels. Univariate and multivariate logistic regression analysis were performed to identify independent risk factors for newborn bone metabolic abnormalities. The results are presented using a forest plot. Statistical significance was set at *p*‐value < 0.05.

## 3 Results

### 3.1 Baseline Characteristics of Included Participants

In total, 197 mothers and matched newborns were included in this study. The prevalence of maternal vitamin K2 deficiency was 38.6%. Among the mothers, 76 and 121 were included in the vitamin K2 deficiency and control groups, respectively. The initial characteristics of the mothers and newborns are summarized in Table 1. Notably, the vitamin K2 deficiency group had significantly lower vitamin K2 levels than the normal group (0.07 ± 0.02 vs. 0.34 ± 0.26 ng/mL; *p <* 0.001). No significant differences were observed between the vitamin K2 deficiency and normal groups regarding age, gestational age, pre‐pregnancy body mass index, weight gain during pregnancy, delivery mode, the prevalence of gestational diabetes mellitus, and gestational hypertension. Similarly, newborn sex, birth weight, and length did not differ between the groups.

**TABLE 1:** Baseline characteristics of the included participants by vitamin K2 level.

Categories	Vitamin K2 level		*p*
	Deficient	Normal	
*n*	76 (38.6%)	121 (61.4%)	
Mother			
Age (year)	32.36 ± 3.67	31.79 ± 4.97	0.472
Gestational age (week)	39.00 ± 0.87	39.29 ± 1.12	0.114
Pre‐pregnancy BMI (kg/m^2^)	21.52 ± 2.84	22.17 ± 3.18	0.223
Weight gain during pregnancy (kg)	15.34 ± 3.73	14.02 ± 5.23	0.087
Delivery mode, *n* (%)			
Vaginal delivery	43 (21.8%)	86 (43.7%)	0.075
Caesarean section	33 (16.8%)	35 (17.7%)	
Gestational diabetes mellitus, *n* (%)			
No	66 (33.5%)	96 (48.7%)	0.292
Yes	10 (5.1%)	25 (12.7%)	
Gestational hypertension, *n* (%)			
No	63 (32.0%)	102 (51.8%)	0.815
Yes	13 (6.6%)	19 (9.6%)	
Vitamin K2 level (ng/mL)	0.07 ± 0.02	0.34 ± 0.26	< 0.001***
Newborn			
Sex, *n* (%)			
Female	33 (16.8%)	60 (30.5%)	0.451
Male	43 (21.8%)	61 (30.9%)	
Birth weight (g)	3272.9 ± 390.25	3305 ± 427.1	0.659
Birth length (cm)	50.16 ± 1.46	50.43 ± 1.45	0.276

*Note:* Values are presented as mean ± SD for continuous variables or a number (percentage) for categorical variables. *p*‐values were calculated using *t‐*tests or chi‐squared tests, as appropriate. ***p < 0.001.

Abbreviations: BMI, body mass index; SD, Standard deviation.

### 3.2 Relationship Between Maternal Vitamin K2 Level and Maternal Bone Metabolism Markers

Table 2 summarizes the differences between the vitamin K2 deficiency and normal groups in terms of maternal and cord blood levels of 25‐OHD, Ca, ALP, OC, and PTH. The average maternal OC levels were higher in the vitamin K2 deficiency group compared to those in the normal group (20.29 ± 8.78 vs. 16.32 ± 5.95 ng/mL, *p* = 0.002). However, no significant differences were observed between the two groups in terms of 25‐OHD, Ca, ALP, or PTH levels (*p* > 0.05).

**TABLE 2:** Relationship between vitamin K2 level and bone metabolic markers.

Characteristics	Vitamin K2 level		*p*
	Deficient	Normal	
*n*	76 (38.6%)	121 (61.4%)	
Mother			
25‐OHD (ng/mL)	20.58 ± 11.01	21.95 ± 12.48	0.515
Ca (mg/dL)	2.20 ± 0.08	2.24 ± 0.21	0.138
ALP (u/L)	141.37 ± 36.02	136.65 ± 49.88	0.551
OC (ng/mL)	20.29 ± 8.78	16.32 ± 5.95	0.002**
PTH (pg/mL)	36.73 ± 22.63	32.84 ± 16.68	0.283
Newborn			
25‐OHD (ng/mL)	9.53 ± 5.11	12.84 ± 7.20	0.002**
Ca (mg/dL)	97.36 ± 7.25	99.05 ± 5.81	0.136
ALP (u/L)	172.89 ± 42.7	160.11 ± 42.16	0.088
OC (ng/mL)	76.53 ± 23.80	61.02 ± 20.80	< 0.001***
PTH (pg/mL)	7.98 ± 7.09	4.82 ± 5.16	0.006**

*Note:* Data are presented as *n* (%) or mean ± standard deviation. *p*‐values were calculated using *t‐*tests; **p < 0.01, ***p < 0.001.

Abbreviations: 25‐OHD, 25‐hydroxyvitamin D; ALP, alkaline phosphatase; Ca, calcium; OC, osteocalcin; PTH, parathyroid hormone.

### 3.3 Relationship Between Maternal Vitamin K2 Level and Cord Blood Bone Metabolism Markers

**FIGURE 1:**
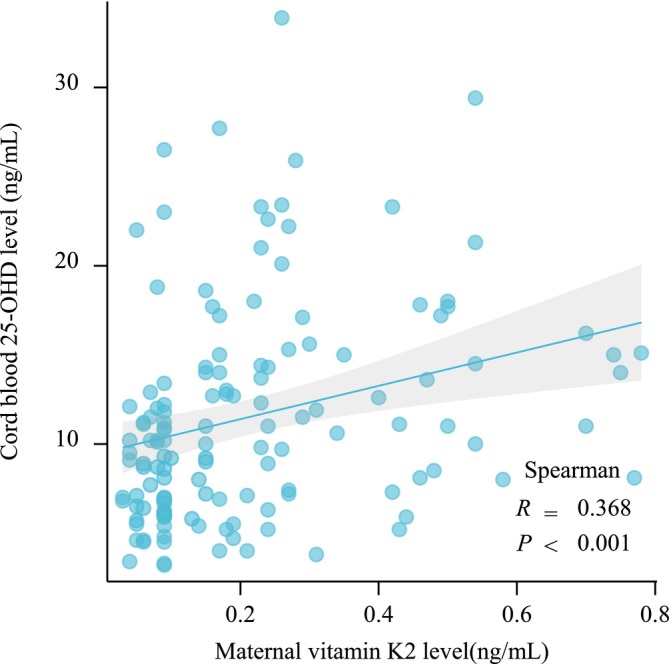
Correlation between maternal vitamin K2 levels and cord blood 25‐OHD levels.

Table 2 shows significant differences in 25‐OHD (9.53 ± 5.11 vs. 12.84 ± 7.20, *p* = 0.002), OC (76.53 ± 23.80 vs. 61.02 ± 20.80, *p* < 0.001), and PTH (7.98 ± 7.09 vs. 4.82 ± 5.16, *p* = 0.006) levels between the deficient and normal groups. Additionally, a positive correlation was observed between maternal vitamin K2 and umbilical cord blood 25‐OHD levels (*r* = 0.368, *p <* 0.001) (Figure 1). Conversely, no differences were observed in Ca and ALP levels between the two groups. These findings indicate a correlation between maternal vitamin K2 levels and bone metabolism markers in the umbilical cord blood. Deficient maternal vitamin K2 levels may contribute to altered 25‐OHD, OC, and PTH levels in mothers and newborns.

### 3.4 Maternal Vitamin K2 as an Independent Risk Factor for 25‐OHD Insufficiency in Newborns

The infants were categorized into two groups based on their 25‐OHD levels: the insufficiency (< 20 ng/mL) and normal (≥ 20 ng/mL) groups. Univariate logistic regression analysis revealed that maternal vitamin K2 levels were associated with a significantly reduced risk of developing neonatal vitamin 25‐OHD insufficiency (OR = 0.121, 95% CI = 0.018–0.806, *p* = 0.029). Maternal 25‐OHD (OR = 0.783, 95% CI = 0.702–0.873, *p <* 0.001) and cord blood Ca (OR = 0.882, 95% CI = 0.808–0.962, *p* = 0.005) levels were also associated with a lower risk of 25‐OHD insufficiency in neonates; conversely, maternal PTH levels were associated with a higher risk (OR = 1.066, 95% CI = 1.016–1.120, *p* = 0.010) (Table 3).

**TABLE 3:** Univariate logistic regression analysis of cord blood 25‐OHD insufficiency.

Characteristics	Total (*N*)	OR (95% CI)	*p*
Mother			
Age	197	0.863 (0.754–1.287)	0.248
Gestational age	197	1.425 (0.899–2.259)	0.132
Pre‐pregnancy BMI	197	0.882 (0.756–1.028)	0.108
Weight gain during pregnancy	197	0.974 (0.878–1.081)	0.626
Vitamin K2	197	0.121 (0.018–0.806)	0.029*
25‐OHD	197	0.783 (0.702–0.873)	< 0.001***
Ca	197	0.724 (0.622–1.068)	0.703
ALP	197	0.999 (0.988–1.009)	0.797
OC	197	1.008 (0.940–1.080)	0.830
PTH	197	1.066 (1.016–1.120)	0.01*
Newborn			
Birth weight	197	1.000 (0.999–1.001)	0.904
Birth length	197	0.962 (0.681–1.359)	0.825
Ca	197	0.882 (0.808–0.962)	0.005**
ALP	197	1.002 (0.990–1.014)	0.799
OC	197	1.020 (0.996–1.046)	0.103
PTH	197	1.173 (0.967–1.422)	0.105

Abbreviations: 25‐OHD, 25‐hydroxyvitamin D; 95% CI, 95% confidence interval; ALP, alkaline phosphatase; BMI, body mass index; Ca, calcium; OC, osteocalcin; OR, odds ratio; PTH, parathyroid hormone.

*p < 0.05, **p < 0.01, ***p < 0.001.

**FIGURE 2:**
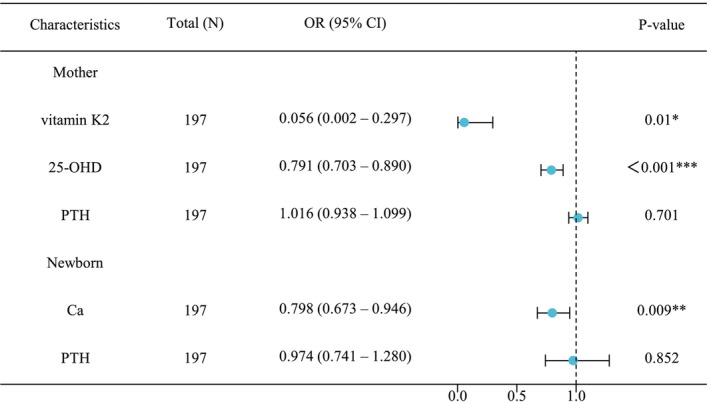
Adjusted odds ratios for calculating risk factors for cord blood 25‐OHD insufficiency using multivariate logistic regression analysis. Differences between data were statistically significant when *p* < 0.05 (*), < 0.01 (**) or < 0.001 (***).

After adjusting for potential confounding factors, multivariate regression analysis was performed, including the significant markers identified above. The forest plot indicated that maternal vitamin K2 (OR = 0.056, 95% CI = 0.002–0.297, *p* = 0.01), maternal 25‐OHD (OR = 0.791, 95% CI = 0.703–0.890, *p <* 0.001), and cord blood Ca (OR = 0.798, 95% CI = 0.673–0.964, *p* = 0.009) levels were significantly associated with a lower risk of 25‐OHD insufficiency in newborns (Figure 2). These findings suggest that maternal vitamin K2 and 25‐OHD levels, as well as cord blood Ca levels, are independent risk factors for neonatal 25‐OHD insufficiency.

## 4 Discussion

Our study found that 38.6% of pregnant women were deficient in vitamin K2, and this deficiency correlated with neonatal bone metabolism markers. Maternal vitamin K2 level was identified as an independent risk factor for 25‐OHD insufficiency in newborns. These findings emphasize the importance of vitamin K2 supplementation in pregnant women.

Vitamin K2 is crucial for maintaining bone strength and contributes to mineralization by activating vitamin K‐dependent proteins that exert their effects outside the liver (Capozzi et al. 2019). The level of vitamin K2 in newborns depends mainly on the level of maternal vitamin K2 (Lippi and Franchini 2011). Consequently, infants of mothers with or at a high risk of vitamin K2 deficiency are also at risk of vitamin K2 deficiency and related complications. Liu et al. (2023) found a relatively high prevalence of vitamin K2 deficiency in neonates (33%) due to limited storage at birth. Our findings revealed a remarkable prevalence of vitamin K2 deficiency among pregnant women during the late stages of pregnancy, with a prevalence as high as 38.6%. Vitamin K2 deficiency is multifactorial and results from increased vitamin K2 demand and inadequate intake (Kozioł‐Kozakowska and Maresz 2022; Zhang et al. 2024). During pregnancy, factors such as hormonal changes, fetal growth and development, and increased blood volume contribute to higher vitamin K2 requirements in pregnant women. Additionally, alterations in diet, nutrition, and gut microbiota can lead to insufficient intake of vitamin K2 (Chatterjee et al. 2023). Therefore, it is imperative to recognize the prevalence of vitamin K2 deficiency in pregnant women during the later stages of pregnancy and encourage them to take vitamin K2 supplements.

Previous studies have primarily focused on the influence of vitamin D on bone health (Dragomir et al. 2024), often overlooking the potential impact of vitamin K2. Recently, there has been growing interest in identifying biomarkers of bone metabolism, such as 25‐OHD, Ca, ALP, OC, and PTH, and their relationship with vitamin K2. Vitamin K2 acts as a cofactor for carboxylation, converting the glutamic acid residues of OC into γ‐carboxyglutamic acid, which is crucial for maintaining normal bone mineralization rates in the skeleton (Zhang et al. 2024). Furthermore, vitamin K2 not only regulates the growth and differentiation of osteoblasts but also prevents their apoptosis and activates genes related to bone formation, including CYP3A4 and MSX2 (Yan et al. 2023). Li et al. (2023) research suggests that higher levels of MK‐7 are linked to the risk of developing osteoporosis and can help diagnose this condition in postmenopausal women. The treatment regimen combining alendronate sodium with vitamin K2 has been shown to significantly lower serum undercarboxylated OC (ucOC) levels and enhance bone density in the lumbar spine and femoral neck in postmenopausal women suffering from rheumatoid arthritis (Suzuki et al. 2012). A meta‐analysis of nine randomized placebo‐controlled clinical trials involving 6853 participants revealed that vitamin K2 supplementation significantly enhanced bone mineral density, reduced fracture risk, and decreased undercarboxylated osteocalcin levels (Zhou et al. 2022). Inaba et al. (2015) reported that a daily intake of ≥ 100 μg MK‐7 with regular meals could improve the γ‐carboxylation status of OC, thereby enhancing bone health. The Japanese Society for Bone and Mineral Research recommends vitamin K2 as a secondary therapy for managing and treating glucocorticoid‐induced osteoporosis (Suzuki et al. 2014). The active form of vitamin D, 25‐OHD, affects bone mineralization and growth by promoting intestinal Ca absorption, directly influencing maternal and infant bone health (Luo et al. 2023). Our findings demonstrated a significant positive correlation between maternal vitamin K2 levels and neonatal 25‐OHD levels, contributing novel insights to the existing literature. Despite vitamin D supplementation during pregnancy, umbilical blood 25‐OHD levels remain insufficient, potentially due to the synergistic effect between vitamin K2 and 25‐OHD. OC is a ca‐binding protein that depends on vitamin K2. It is produced and released by non‐proliferating osteoblasts and serves as a non‐collagenous protein in the bone matrix (Wang, Mazur et al. 2021; Wang, Zhang et al. 2021). The active form of OC, ucOC, binds to Ca, and hydroxyapatite, reducing abnormal crystallization and preserving normal mineralization (Wang and Ma 2023). Previous studies found that MK‐4 reduced ucOC levels and enhanced bone health in ovariectomized mice (Wang, Mazur et al. 2021; Wang, Zhang et al. 2021). Our research revealed that pregnant women with vitamin K2 deficiency had higher OC concentrations compared to those in the normal group, as did their newborns. The lack of γ‐carboxylation in OC, caused by a deficiency of vitamin K2, prevents calcium ions from binding to the bone matrix. Additionally, PTH, produced by the chief cells of the parathyroid gland, plays a vital role in balancing calcium and phosphorus levels and regulating bone metabolism (Tamura et al. 2016). Our study indicated that newborns in the maternal vitamin K2 deficiency group have significantly higher levels of PTH compared to those in the normal group. Low blood Ca levels trigger PTH secretion (Yamashita et al. 2024), and this association may be linked to deficient vitamin K2 levels. In summary, these findings indicate a significant relationship between maternal vitamin K2 levels and bone metabolism markers in both mothers and neonates. Our results support the hypothesis that adequate maternal vitamin K2 levels are crucial for optimal neonatal bone health.

Vitamin D plays a crucial role in promoting bone health and development (Mendes et al. 2022). Insufficient 25‐OHD levels in newborns are associated with an increased risk of various skeletal disorders and poor bone health (Dan et al. 2023). Several factors contribute to 25‐OHD insufficiency in neonates, including inadequate maternal vitamin D intake, limited sun exposure, and genetic and environmental factors (Abrams 2020). However, the relationship between maternal vitamin K2 levels and 25‐OHD insufficiency in newborns has not been extensively investigated. In our study, the univariate analysis results showed that maternal vitamin K2, maternal 25‐OHD, and cord blood Ca levels were associated with a lower risk of neonatal 25‐OHD insufficiency. Conversely, maternal PTH level has been identified as a risk factor for neonatal 25‐OHD insufficiency. Furthermore, in the multivariate analysis, after adjusting for confounding factors, maternal vitamin K2, maternal 25‐OHD, and cord blood Ca levels remained independent risk factors for 25‐OHD insufficiency in newborns. These results demonstrate an independent association between maternal vitamin K2 and neonatal 25‐OHD insufficiency, confirming the importance of maternal vitamin K2 as a standalone risk factor. Thus, adequate maternal vitamin K2 levels during pregnancy may protect neonates from 25‐OHD insufficiency.

This study had some limitations. First, vitamin K2 intake largely depends on dietary preferences and habits. Our study was conducted at a single center, and the generalizability of our findings to other regions or populations with different dietary patterns may be limited. Larger multicenter studies are required to validate these findings. Second, this study lacked a long‐term follow‐up; therefore, it was impossible to further explore the prognosis of vitamin K2 deficiency in children. Third, the molecular pathways and specific mechanisms by which vitamin K2 regulates bone metabolism remain unclear.

## 5 Conclusions

This study is the first to investigate the relationship between maternal vitamin K2 levels and bone metabolism markers in newborns. These findings illustrate that vitamin K2 deficiency is prevalent among pregnant women in the late stages of pregnancy and is an independent risk factor for 25‐OHD insufficiency in newborns. Our study highlights the potential importance of monitoring and supplementing vitamin K2 levels during pregnancy to promote optimal skeletal development in newborns. Further research is needed to explore the levels of vitamin K2 during different stages of pregnancy and establish the recommended daily intake of vitamin K2.

## Author Contributions

Xuejing Liu: data curation (equal), investigation (lead), methodology (equal), writing – original draft (lead). Shuo Wang: methodology (equal), validation (equal). Han Chen: software (equal), visualization (equal). Nianfeng Qian: formal analysis (equal), validation (equal). Lina Wu: data curation (equal), validation (equal). Yingnuo Liu: formal analysis (equal). Zhaoxi Hou: methodology (equal). Yueting Bai: software (equal). Hongqing Jiang: conceptualization (lead), project administration (lead), supervision (lead), funding acquisition (lead). All authors have read and agreed to the published version of the manuscript.

## Ethics Statement

This study was conducted following the Helsinki Declaration and approved by the Research Ethics Committee of Haidian Maternal and Child Health Hospital (No. SB2024‐01). All included pregnant women were informed about the details, benefits, and obligations of this study. Written informed consent was obtained from all participants before the study.

## Conflicts of Interest

The authors declare no conflicts of interest.

## Data Availability

All supporting data used and analyzed in this study are available from the corresponding author upon reasonable request.
